# Implementation of a pooled surveillance testing program for asymptomatic SARS-CoV-2 infections in K-12 schools and universities

**DOI:** 10.1016/j.eclinm.2021.101028

**Published:** 2021-07-17

**Authors:** Rachelle P. Mendoza, Chongfeng Bi, Hui-Ting Cheng, Elmer Gabutan, Guillerre Jan Pagapas, Nadia Khan, Helen Hoxie, Stephen Hanna, Kelly Holmes, Nicholas Gao, Raychel Lewis, Huaien Wang, Daniel Neumann, Angela Chan, Meril Takizawa, James Lowe, Xiao Chen, Brianna Kelly, Aneeza Asif, Keena Barnes, Nusrat Khan, Brandon May, Tasnim Chowdhury, Gabriella Pollonini, Nourelhoda Gouda, Chante Guy, Candice Gordon, Nana Ayoluwa, Elvin Colon, Noah Miller-Medzon, Shanique Jones, Rauful Hossain, Arabia Dodson, Meimei Weng, Miranda McGaskey, Ana Vasileva, Andrew E. Lincoln, Robby Sikka, Anne L. Wyllie, Ethan M. Berke, Jenny Libien, Matthew Pincus, Prem K. Premsrirut

**Affiliations:** a Mirimus Inc, 760 Parkside Ave. Suite 206, Brooklyn, NY 11226, USA; b Department of Pathology, SUNY Downstate Health Sciences University, 450 Clarkson Ave., Brooklyn, NY 11226, USA; c Department of Cell Biology, SUNY Downstate Health Sciences University, 450 Clarkson Ave., Brooklyn, NY 11226, USA; d MedStar Sports Medicine Research Center, MedStar Health Research Institute, 2900 S Hanover St., Baltimore, MD 21225, USA; e Department of Rehabilitation Medicine, Georgetown University Medical Center, 3800 Reservoir Rd NW, Washington, DC 20007, USA; f Minnesota Timberwolves, 600 Hennepin Ave, Minneapolis, MN 55403, USA; g Department of Epidemiology of Microbial Diseases, Yale School of Public Health, 60 College St, New Haven, CT 06510, USA; h OptumLabs, UnitedHealth Group, 12700 Whitewater Dr, Minnetonka, MN 55343 USA

**Keywords:** SARS-CoV-2, Pooled surveillance testing, Asymptomatic infections, K-12 schools

## Abstract

**Background:**

The negative impact of continued school closures during the height of the COVID-19 pandemic warrants the establishment of cost-effective strategies for surveillance and screening to safely reopen and monitor for potential in-school transmission. Here, we present a novel approach to increase the availability of repetitive and routine COVID-19 testing that may ultimately reduce the overall viral burden in the community.

**Methods:**

We implemented a testing program using the SalivaClear࣪ pooled surveillance method that included students, faculty and staff from K-12 schools (student age range 5–18 years) and universities (student age range >18 years) across the country (Mirimus Clinical Labs, Brooklyn, NY). The data analysis was performed using descriptive statistics, kappa agreement, and outlier detection analysis.

**Findings:**

From August 27, 2020 until January 13, 2021, 253,406 saliva specimens were self-collected from students, faculty and staff from 93 K-12 schools and 18 universities. Pool sizes of up to 24 samples were tested over a 20-week period. Pooled testing did not significantly alter the sensitivity of the molecular assay in terms of both qualitative (100% detection rate on both pooled and individual samples) and quantitative (comparable cycle threshold (Ct) values between pooled and individual samples) measures. The detection of SARS-CoV-2 in saliva was comparable to the nasopharyngeal swab. Pooling samples substantially reduced the costs associated with PCR testing and allowed schools to rapidly assess transmission and adjust prevention protocols as necessary. In one instance, in-school transmission of the virus was determined within the main office and led to review and revision of heating, ventilating and air-conditioning systems.

**Interpretation:**

By establishing low-cost, weekly testing of students and faculty, pooled saliva analysis for the presence of SARS-CoV-2 enabled schools to determine whether transmission had occurred, make data-driven decisions, and adjust safety protocols. We provide strong evidence that pooled testing may be a fundamental component to the reopening of schools by minimizing the risk of in-school transmission among students and faculty.

**Funding:**

Skoll Foundation generously provided funding to Mobilizing Foundation and Mirimus for these studies.

**Research in context**

**Evidence before this study**

We started to pursue pooled saliva testing as a pilot project in May 2020 when there was no FDA-approved saliva testing for the diagnosis of COVID-19, and one pooling strategy published by WHO. Prior to writing the paper, we searched PubMed for all manuscripts containing the keywords “COVID-19 Pooled Surveillance” or “COVID-19 Pooled Testing.” Although there are many publications discussing pooling, most of these studies consist of predictive modeling, proof-of-concept testing protocols and strategic analyses.

**Added value of this study**

To our knowledge, there are no other published research articles showing the results of a real-life, large scale, K-12 school-based pooled saliva testing to date. We devised and implemented a high sensitivity RT-PCR, saliva-based, pooled surveillance strategy in 111 schools and universities and tested nearly 300,000 samples collected from 111 K-12 schools and universities in a 20-week period. By performing surveillance testing on entire populations, schools were able to keep their positivity rates low (0·2% on average). Through our work, we demonstrate the power of surveillance testing, whereby cases can be identified and isolated before an outbreak occurs, often identifying infected individuals with trace viral loads days before symptom onset.

**Implications of all the available evidence**

The closure of schools has affected more than 50 million children in the USA, not only negatively affecting learning aptitude but also causing severe negative psychosocial impacts as well, in which the full ramifications of these events will remain unknown for years to come. Our solution uses a saliva-based RT-PCR pooled testing approaching that is highly sensitive and scalable, enabling rapid turnaround time (within 24 h) for tens of thousands of samples in parallel. We believe that with mass testing, along with social distancing and mass wearing, we can reopen schools safely and minimize transmission rates within schools through early identification followed by proper quarantine procedure in order to prevent outbreaks.

Alt-text: Unlabelled box

## 1 Introduction

The number of daily tests for SARS-CoV-2 in the United States increased from approximately 400,000 in the summer of 2020, to 1 million daily tests in September, to 2 million in January 2021 [1]. Despite this increase, access to affordable testing remains limited. New strategies for surveillance and screening are needed to increase the availability of testing and to reduce the overall viral burden in the community while monitoring for virus resurgence. Since asymptomatic and pre-symptomatic cases are believed to be the main drivers of SARS-CoV-2 spread [2], surveillance testing of asymptomatic individuals to screen for the presence of SARS-CoV-2 has been advocated by public health officials for interrupting chains of transmission. Despite the advent of COVID −19 vaccines, continued testing is required to monitor the spread of new SARS-CoV-2 variants and for assessing the efficacy of current vaccination strategies [3]. While reverse transcription–polymerase chain reaction (RT-PCR) remains the gold standard method for detecting SARS-CoV-2, cost-effective strategies are desperately needed to permit mass testing efforts.

Pooling samples for surveillance testing for SARS-CoV-2 is one effective approach for minimizing the resources required and providing a “multiplier” for existing testing frameworks [4], [5], [6], [7]. This approach for testing individuals is not a new idea. Pooled testing methods were integral during HIV epidemics [8] to reduce the costs of testing. In addition, pooled testing of blood donations has been routinely used for hepatitis *B* virus, hepatitis *C* virus and HIV screening in blood banks [9]. While many pooled testing strategies have been proposed to combat the limited testing capacity during the recent pandemic, few have been successfully implemented into testing labs at a scale that can impact the community at large. Importantly, those that have, have primarily focused on swabs, which have been shown to miss nearly 15% of cases when self-collected and or miss samples with low viral loads [10,11]. Saliva in comparison, can be reliably self-collected, reducing the burden on healthcare workers and arrives in a format that can readily be pooled [6,7,12]. Furthermore, a recent study demonstrated that SAR-CoV-2 was detected in saliva using RT-PCR 1.5–4.5 days before the viral load could be detected in a paired nasal swab [13], which may explain how an independent group observed a higher efficiency of detecting asymptomatic infected individuals using saliva pooling (pool size of up to 12) when compared to nasal swabbing methods [14]. Although there are many publications discussing pooling, most of these studies consist of predictive modeling, proof-of-concept testing protocols and strategic analyses [12,15,16]. One modeling study predicted that saliva-based sample and pooling samples could reduce cost by 40% and personnel by 20% [15]. To our knowledge, there are no other published research articles showing the results of a real-life, large scale, K-12 school-based pooled saliva testing to date.

Here, we analyzed the reliability and cost-minimization capacity of real–world implementation of pooled testing in elementary-to-high (K-12) schools and universities to provide an affordable approach to surveillance testing of entire school populations.

## 2 Methods

### 2.1 Study design

This is a quality improvement program that aims to increase the capacity for COVID-19 testing using a pooled, high analytical sensitivity, low-cost surveillance testing method. We analyzed the reliability and cost-efficiency of the program compared to published individual testing protocols.

### 2.2 Ethical approval

The SUNY Downstate Health Sciences University Institutional Review Board (IRB) reviewed and approved the study protocol (IRB #1,232,938–3).

### 2.3 Study subjects

Samples were obtained from K-12 and university students, faculty and staff members attending educational institutions participating in the SalivaClear™ (Mirimus Clinical Labs, Brooklyn, NY) pooled SARS-CoV-2 surveillance from August 27, 2020 to January 13, 2021. SalivaClear™ is a pooled testing program that utilizes a laboratory-developed saliva-based, quantitative, real time RT-PCR test surveillance model adopted by numerous school districts in four northeast states. Written informed consent for COVID-19 testing was obtained from each participant from all institutions prior to the start of the surveillance program.

### 2.4 Specimen collection and processing

**Fig. 1:**
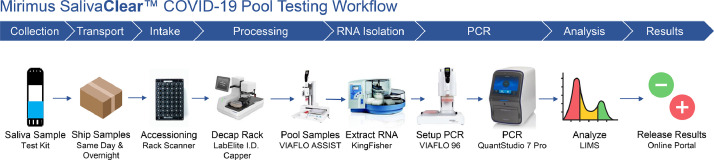
Flow diagram of the process from specimen collection to testing and result analysis.

Students, faculty and staff were tested 1,2 times per week using a twenty-four to one (24:1) SalivaClear™ pooled testing program. The specimen collection was performed under supervision of a trained representative from each organization. The training of the specimen collection supervisor was provided by laboratory professionals and involved a video tutorial followed by a live demonstration and FAQs session. Subjects were asked to collect saliva by passively drooling through a saliva collection device inserted into a 2 ml dry, sterile tube, each with two barcodes for accurate specimen identification. The saliva specimens were grouped by organizations’ “common exposure environment” guidelines in sets of up to 24 individuals sharing the same exposure environment (i.e., same classroom, same office). The specimens were packed and shipped according to current CDC guidelines on packing and shipping of infectious specimens. The median shipment time was 12 h (range: 8–16 h). Fig. 1 shows a flow diagram of the whole process, from specimen collection to testing and result reporting.

### 2.5 Pooling of saliva

The saliva specimens were received in volumes ranging from <0.5 to 1.5 ml. Any specimen less than 0.5 ml was rejected. Specimens that were solidified and/or containing any solid or foreign material were also excluded from testing. Each set of 24 specimens was scanned into the accessioning system that creates a permanent snapshot of the location of individual tubes within that set. A designated “pool tube” was scanned with the 24 tubes. The individual tubes were automatically decapped and placed on an automated liquid handler. To decrease the viscosity of the saliva specimens, 50 µl of 0·4 M 1,4-dithiothreitol (DTT) was added and mixed with the saliva to facilitate pipetting. Following mixing, 200 µl of saliva from each individual specimen was pipetted into a sterile reservoir and mixed thoroughly to create a homogenous solution with up to 4.8 ml in total volume. A total of 3.5 ml of this pooled solution was transferred to the designated 4-ml pre-barcoded “pool tube.”

### 2.6 RNA extraction and SARS-CoV-2 assay

**Fig. 2:**
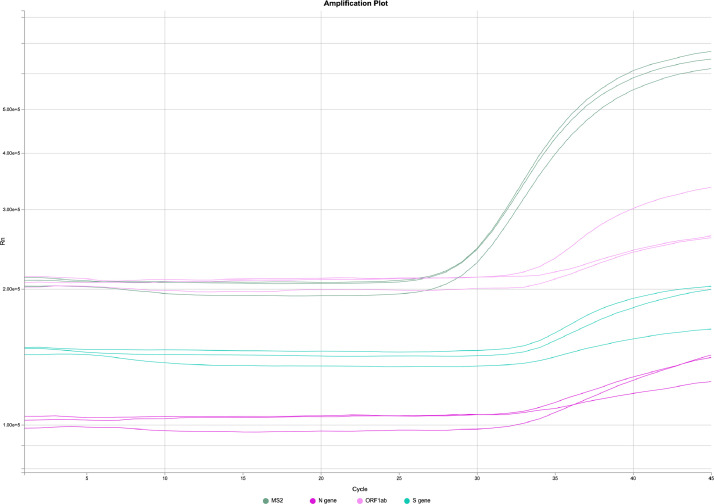
A graph of a quantitative RT-PCR amplification from a positive pooled sample (24 samples) with a Ct value of 37.02, 36.37 and 36.04 for the *N* gene, ORF1ab and *S* gene targets, respectively. Each line indicates the amplification curve for one replicate.

RNA extraction on pooled and individual saliva specimens was conducted using the MagMAX Viral/Pathogen Nucleic acid isolation kit automated on the KingFisher Flex Purification system (Thermo Fisher Scientific, Waltham, MA) following manufacturer's protocol. The RT-PCR including cDNA synthesis and PCR amplification of the target sequences was performed in either triplicate or duplicate PCR reactions on pools or individual specimens, respectively, using the QuantStudio 7 Pro-Real-Time Flex PCR system (Applied Biosystems, Foster City, CA). The assay utilized the primer and probe sets included in the TaqPath COVID-19 Combo Kit (FDA EUA on March 13, 2020) (Thermo Fisher Scientific, Waltham, MA). These primer and probe sets were designed to amplify and detect three regions of the SARS-CoV-2 single stranded RNA genome: the ORF1ab, *N* and *S* genes. In the same assay, MS2 phage RNA and a primer targeting human RNase *P*, Hs_RPP30 (RP) were used as internal positive controls for RNA extraction and RT-PCR. The amplification of either the MS2 or RNase P validates the assay. The data was analyzed by Applied Biosystems Design and Analysis Software version 2·3·4 which is authorized under FDA EUA for interpretation of individual diagnostic tests only, therefore the software was used only for guidance on pooled specimens. For individual specimens, detection of 2 or more gene targets with cycle threshold (Ct) values of at most 37 indicated a positive result, whereas detection of only 1 of the three viral gene targets was deemed inconclusive. For pooled specimens, amplification of a single target with Ct values of at most 40 (Fig. 2) was deemed positive and resulted in reflex testing for deconvolution to identify the positive specimen(s) within that pool. Because triplicate PCR reactions were performed, there was a potential for 9 amplifications (3 amplifications for 3 targets). Even a single amplification with a Ct <40 and Cq confidence score >0.8 triggered reflex to increase the chance of detection of low viral load samples. Results were entered into an electronic health records portal and reported to the individual schools and state departments of health.

The limit of detection (LOD) of the RT-PCR assay was validated using whole heat-inactivated SARS-CoV-2 virus (ATCC® VR-1986HK™, Manassas, VA) spiked into known negative saliva. A preliminary range finding experiment was performed by testing three independent RNA extractions followed by RT-PCR of each RNA extraction in triplicate reactions, using a 2-fold serial dilution of quantitated SARS-CoV-2 spiked saliva. Viral concentrations ranging from 100,000 to 500 viral genome copy equivalents per ml (GCE/ml) of specimen were tested. To go below the LOD, additional samples with viral concentrations ranging from 5000 to 20 GCE/ml were generated and tested. The LOD was determined to be the lowest concentration at which at least 100% of replicates were detected. The preliminary LOD observed in this initial experiment was subsequently confirmed by testing 20 RNA extraction replicates at that concentration, using whole viral genomic RNA, spiked heat-inactivated, negative saliva.

The LOD for a pooled specimen was determined by the viral load that triggered reflex testing in 20 out of 20 replicates. Using a 2-fold serial dilution of quantitated whole heat-inactivated SARS-CoV-2 virus spiked into individual negative human saliva, individual samples with viral concentrations from 5000 to 20 GCE/ml of specimen were generated. Two hundred (200) μl of each of these spiked individual samples were combined with a corresponding number of 200 μl individual negative saliva samples to make pools of 8, 16, 24, 36 or 48. The pools were mixed thoroughly in a sterile reservoir. Two hundred (200) μl of each pooled specimen was tested in three independent RNA extractions followed by RT-PCR of each RNA extraction, performed in triplicate reactions. The LOD of the pooled specimen was determined to be the lowest concentration of individual saliva specimen in the highest number of pool size with 100% triggering reflex to individual testing.

### 2.7 Statistical methods

Statistical analyses were performed in RStudio v1·3·959 (http://www.rstudio.com/), using R v4.0.2 (https://www.r-project.org/) and data visualization was performed by R package ggplot2 (https://ggplot2.tidyverse.org) [17,18]. To determine the reliability of pooled saliva surveillance testing, we performed LOD determination in individual and pooled saliva samples following FDA guidelines. We then compared the detection capacity of saliva testing with the existing reference testing modality (nasal/nasopharyngeal swab) by analyzing concurrently collected saliva and swab samples from 120 individuals. The agreement between the two tests was analyzed using kappa coefficient analysis. The qualitative results were summarized using descriptive statistics and the difference in Ct values between the two diagnostic modalities were assessed using Wilcoxon signed-rank test. We also compared our data with the community data and assessed for trend similarity. An outlier detection analysis was conducted to validate that our dataset followed a similar trend, with respect to anomalies, as gold standard epidemiological surveillance datasets, such as those published by the CDC (https://www.cdc.gov/coronavirus/2019-ncov/covid-data/data-visualization.htm). While such a comparison does not provide insights in and of itself, demonstrating that the trends are comparable suggests that we are conducting surveillance at a sufficient scale to replicate general population trends, at least with respect to replicating anomalies. The anomalies investigated are the spike in positivity rates following mass gathering events on October 31st, December 25th, and December 31st, corresponding to the holidays of Halloween, Christmas, and New Years in the US.

Our underlying distribution for positivity is believed to be non-normal due to the number of dates where 0 positives were detected, skewing the distribution. Furthermore, the assumed true distribution provided by CDC data may or may not be multimodal, but there were several shifts in the peaks. To avoid the assumptions associated with normal distributions, a non-parametric analytical method was used. There were several dates across the dataset with a low testing volume (< 500), which do not meet a threshold to determine true positivity rate. These dates are outliers with high positivity rates and are believed to be erroneous, due to low volumes, as opposed to a reflection of testing realities, as indicated by the John Hopkins Coronavirus Resource Center (https://coronavirus.jhu.edu/testing/testing-positivity). To avoid these possibly erroneous outliers misdirecting the results of the analysis, all dates with less than 500 tests performed were removed. Our data is a time series, hence non-parametric methods of investigating outliers in a time series were investigated and the modified Z score outlier detection method outlined by Iglewicz and Hoaglin (1993) were selected [19]. The method calculated the modified Z score as detailed below and, if the modified Z score was greater than 3·5, that observation was presumed to be an outlier. Time series data was impacted by local time variations, otherwise known as seasonality, and these variations are a critical aspect of investigating infectiousness through an epidemiological lens [20]. To avoid seasonality interference regarding mass gathering date outlier detection, the dates included in the analysis for each mass gathering date were restricted. For October 31, only the dates going one month prior and one month after were included, ranging from October 1 to December 1. For December 25 and December 31 our data only extends until January 13 so, to maintain the two-month evaluation range, we used data from November 13 extending until January 13. For the October 31 dataset, this restricted the number of dates, or data points, to 39. For December 25 and December 31, or Christmas and New Year, this restricted the number of dates, or data points, to 31.

A cost-minimization analysis was performed by comparing the unit cost of the surveillance testing with the unit cost of the reference testing modalities using published data. We also estimated the total cost averted by the ability of the surveillance testing to detect early infection among the asymptomatic population and prevent transmission among close contacts within an organization. The raw data set for this study is available on a secure server hosted by Mirimus Clinical Labs, Brooklyn, NY.

No sample size computation was performed due to lack of reliable published data on the rate of COVID-19 infection in asymptomatic student population. This is an exploratory study on the infection rate on K-12 and university population. The writing of this manuscript was performed with strict adherence to STROBE guidelines (https://www.equator-network.org/reporting-guidelines/strobe/).

### 2.8 Role of the funding source

The funding source provided financial support to proceed with this project. The Skoll Foundation did not have any role in the study design, collection, analysis, interpretation of data, writing of the report and in the decision to submit the paper for publication.

## 3 Results

Our goal was to develop a reliable and cost-efficient screening and surveillance COVID-19 testing strategy to be utilized in settings where frequent testing among asymptomatic individuals may be required to prevent early transmission. We first determined the limit of detection (LOD) of the RT-PCR assay using validated whole heat-inactivated SARS-CoV-2 virus spiked into known negative saliva. The preliminary range finding showed consistent detection of two to three viral gene targets at the lowest concentration of 313 virus GCE/ml of individual sample (Supplementary Table 1). Confirmation of the preliminary LOD (313 GCE/ml) and a level higher (625 GCE/ml) were analyzed in an additional 20 independent RNA extractions followed by RT-PCR. At least two targets were detected in 20 out of 20 replicates (detection rate of 100%) in the viral concentration level of 625 GCE/ml (Table 1). This was the identified LOD for individual samples. Similar concentrations of inactivated whole virus were spiked to individual RT PCR-negative saliva specimens, each pooled with 7–47 RT-PCR-negative individual saliva specimens to determine the LOD in pooled specimens. A viral load of 1250 GCE/ml spiked in a 200 μl single sample pooled with 23 RT-PCR-negative saliva samples (200 μl each) triggered reflex for individual testing in 95.0% of the 20 replicates (Table 2; Supplementary Table 2). In accordance with the FDA guidelines on LOD determination, detection of the virus in at least 95% of the 20 replicates defines the LOD level. Hence, 1250 viral GCE/ml was the LOD for the pooled specimen sample.

**Table 1:** Limit of detection (LOD) confirmation using 625 and 313 viral genome copy equivalents per ml (GCE/ml) of individual specimens. A total of twenty independent RNA extractions were performed, followed by RT-PCR reactions, each performed in triplicate for each RNA extraction.

Viral Conc	Replicate	Mean Ct (SD; *N* = 3)					Interpretation	% Pos
		ORF1ab	N	S	MS2	RP		
625 GCE/ml (3.1 GCE/rxn)	1	28.21(1.8)	25.66(NA)	ND	28.7(0.68)	25.58(0.05)	Positive	100%
	2	31.09(0.15)	ND	29.79(NA)	29.44(1.23)	25.28(0.08)	Positive	
	3	30.69(0.59)	30.86(NA)	28(NA)	29.9(0.7)	25.41(0.09)	Positive	
	4	31.26(0.95)	ND	29.82(NA)	29.74(0.65)	25.43(0.22)	Positive	
	5	30.75(0.97)	ND	29.85(0.64)	29.99(0.84)	25.6(0.06)	Positive	
	6	30.56(0.32)	30.12(NA)	29.99(0.19)	28.95(0.31)	25.13(0.65)	Positive	
	7	31.75(1.04)	ND	29.31(2.36)	29.16(0.76)	25.68(0.13)	Positive	
	8	30.07(1.41)	31.46(0.81)	ND	28.64(0.44)	25.67(0.29)	Positive	
	9	31.38(0.57)	28.72(0.96)	29.89(0.05)	28.89(0.72)	25.33(0.04)	Positive	
	10	31.12(0.26)	31.38(NA)	29.53(NA)	29.44(0.68)	25.42(0.12)	Positive	
	11	31.01(0.85)	ND	30.7(0.25)	29.82(0.69)	25.26(0.13)	Positive	
	12	30.66(0.92)	ND	29.58(NA)	29.71(0.55)	25.23(0.1)	Positive	
	13	30.05(1.04)	ND	28.83(NA)	30.03(0.58)	25.45(0.23)	Positive	
	14	30.78(0.01)	ND	30.43(NA)	29.87(0.66)	25.85(0.06)	Positive	
	15	29.07(3.14)	ND	29.4(NA)	29.1(0.71)	25.55(0.27)	Positive	
	16	30.6(0.07)	31.15(NA)	28.29(0.47)	28.88(0.69)	25.62(0.08)	Positive	
	17	30.83(NA)	ND	30.26(NA)	28.76(0.56)	25.43(0.09)	Positive	
	18	31.2(0.81)	30.74(NA)	ND	29.38(0.72)	25.62(0.07)	Positive	
	19	32.07(0.7)	ND	30.91(NA)	29.58(0.78)	25.61(0.08)	Positive	
	20	31.75(0.79)	32.07(NA)	ND	29.35(0.88)	25.17(0.22)	Positive	
313 GCE/ml (1.6 GCE/rxn	1	30.22(0.94)	30.06(NA)	30.79(0.53)	29.72(0.58)	25.37(0.19)	Positive	55%
	2	31.15(0.6)	28.85(0.93)	ND	29.63(0.83)	25.49(0.1)	Positive	
	3	31.22(0.05)	28.9(NA)	ND	29.26(0.76)	25.54(0.1)	Positive	
	4	30.06(0.69)	34.79(NA)	ND	28.29(0.6)	25.07(0.41)	Positive	
	5	30.49(NA)	ND	30.79(NA)	28.7(0.59)	25.32(0.05)	Positive	
	6	ND	ND	ND	29.35(0.77)	25.46(0.1)	Negative	
	7	31.55(1.28)	ND	ND	29.4(0.91)	25.36(0.07)	Inconclusive	
	8	30.45(0.33)	ND	29.7(NA)	29.14(0.82)	25.02(0.11)	Positive	
	9	31.64(0.78)	ND	31.66(NA)	29.43(1.04)	25.37(0.05)	Positive	
	10	30.55(0.88)	ND	30.55(NA)	29.38(0.68)	25.59(0.13)	Positive	
	11	31.07(0.83)	ND	ND	28.94(0.87)	25.54(0.19)	Inconclusive	
	12	31.28(1.69)	ND	ND	28.48(0.92)	25.66(0.07)	Inconclusive	
	13	31.14(1.36)	ND	ND	28.34(0.82)	25.21(0.07)	Inconclusive	
	14	29.81(0.14)	30.19(NA)	27.77(NA)	29.23(0.94)	25.38(0.05)	Positive	
	15	30.99(1.42)	ND	30.17(NA)	29.41(0.97)	25.14(0.06)	Positive	
	16	32.29(0.39)	ND	ND	29.39(1.04)	25.27(0.13)	Inconclusive	
	17	29.59(0.33)	30.58(0.65)	29.85(NA)	29.25(0.89)	25.3(0.1)	Positive	
	18	30.56(1.45)	ND	28.59(NA)	28.98(0.79)	25.42(0.08)	Positive	
	19	29.94(0.28)	ND	ND	29.12(0.9)	25.53(0.12)	Inconclusive	
	20	30.51(0.34)	ND	ND	28.36(0.87)	25.33(0.03)	Inconclusive	

*N* = number of RNA extractions; RP = Hs_RPP30 primer for targeting human RNase *P*.

**Table 2:** Summary of LOD preliminary range finding and confirmation in pooled specimens using known concentrations of ATCC VR-1986HK whole inactivated virus spiked into individual negative saliva. The stock concentration provided by ATCC was 4.2 × 10^5^ GCE/µL. For each RNA extraction, 200 µL of each sample was used.

Viral Conc	Pool size	N	Mean Ct (SD; *N* = 3)					Result	% Triggering Reflex
			ORF1ab	N gene	S gene	MS2	RP		
1250 GCE/ml (6.3 GCE/rxn)	1	1	29.96(0.3)	28.37(0.34)	24.94(4.28)	28.67(1.06)	23.29(0.45)	Positive	100%
		2	29.99(0.43)	28.51(1.44)	25.75(5.33)	28.11(0.47)	23.04(0.04)	Positive	
		3	30.67(1.01)	28.86(2)	26.36(2.34)	28.27(0.28)	22.88(0.16)	Positive	
	8	1	32.83(0.62)	30.18(0.31)	30.25(0.75)	27.9(0.5)	23.19(0.08)	Positive	100%
		2	31.97(0.64)	30.4(0.1)	27.73(1.87)	28.24(0.41)	23.32(0.11)	Positive	
		3	32.36(1.18)	30.11(2.73)	25.88(4.4)	28.51(0.4)	23.23(0.07)	Positive	
	16	1	33.37(0.6)	31.89(1.04)	33.27(NA)	27.98(0.57)	23.02(0.07)	Positive	100%
		2	32.28(NA)	ND	ND	28.4(0.93)	23.12(0.02)	Inconclusive	
		3	ND	31.38(NA)	28.46(NA)	28.9(0.59)	23.19(0.14)	Positive	
	24	1	32.65(1.13)	31.87(NA)	31.05(NA)	28.16(0.92)	23(0.04)	Positive	95.83%
		2	33.74(0.03)	30.8(NA)	ND	28.54(0.86)	23.08(0.04)	Positive	
		3	33.76(0.43)	ND	30.01(0.9)	29.22(0.37)	23.03(0.11)	Positive	
		4	31.29(NA)	30.5(NA)	ND	27.23(0.97)	22.72(0.05)	Positive	
		5	32.58(0.12)	32.47(NA)	ND	27.04(0.7)	22.79(0.22)	Positive	
		6	32.96(0.6)	ND	24.91(NA)	27.34(1.11)	22.83(0.07)	Positive	
		7	33.76(NA)	ND	ND	27.63(0.3)	22.95(0.23)	Inconclusive	
		8	30.88(NA)	ND	ND	27.79(0.77)	23.17(0.13)	Inconclusive	
		9	33.62(NA)	32.82(NA)	32.23(0.12)	27.83(0.37)	22.7(0.15)	Positive	
		10	33.52(NA)	31.03(NA)	ND	28.24(0.52)	23.3(0.06)	Positive	
		11	32.73(NA)	ND	27.8(NA)	27.89(0.26)	22.98(0.12)	Positive	
		12	33.31(1.73)	30.85(NA)	30.5(NA)	28.03(0.63)	23.1(0.03)	Positive	
		13	32.36(NA)	31.92(NA)	ND	27.87(0.44)	22.96(0.05)	Positive	
		14	32.53(NA)	31.87(0.29)	30.92(NA)	28.24(0.09)	23.09(0.13)	Positive	
		15	32.6(0.37)	31.34(1.44)	27.65(3.36)	27.81(0.22)	22.8(0.18)	Positive	
		16	32.54(NA)	ND	ND	28.37(0.57)	23.05(0.02)	Inconclusive	
		17	ND	ND	29.7(NA)	27.94(0.3)	23.14(0.1)	Inconclusive	
		18	ND	ND	ND	28.21(0.67)	23.05(0.03)	Negative	
		19	33.07(NA)	ND	30.85(1.38)	27.91(0.21)	23.22(0.11)	Positive	
		20	32.86(NA)	ND	32.46(NA)	27.79(0.65)	23.31(0.14)	Positive	
	36	1	34.12(NA)	29.38(NA)	ND	28.36(0.56)	23.02(0.05)	Positive	100%
		2	33.87(NA)	30.69(2.08)	25.13(NA)	28.6(0.73)	23.06(0.08)	Positive	
		3	33.56(NA)	25.9(0.05)	ND	28.13(1.09)	22.95(0.04)	Positive	
	48	1	33.78(NA)	31.32(NA)	ND	28.05(0.76)	23.1(0.04)	Positive	66.67%
		2	ND	32.12(NA)	31.42(NA)	28.41(0.49)	23.35(0.05)	Positive	
		3	ND	ND	ND	27.86(1.17)	23.09(0.05)	Negative	

The pooling method was further evaluated using clinical samples. To demonstrate that individual clinical samples are detectable in pools, clinical samples with Ct values ranging from 27 to 31 were used to generate pools of 8, 16, 24, 36 and 48, using 200 µL of the known positive sample combined with negative saliva matrix. There was consistent detection and 100% triggering reflex for pools of 24 (Table 3).

**Table 3:** Confirmation of the LOD using pooled clinical samples. Known positive samples with a Ct values ranging from 27 to 31 were used to generate pools of 8, 16, 24, 36 or 48 using 200 µL of known positive sample combined with negative saliva matrix. A total of 100 µL or 200 µL is used for RNA extraction for individual samples and pooled samples, respectively.

			Mean Ct values (SD; *N* = 3)				
Sample No.	Pool Size	N	MS2	N gene	ORF1ab	S gene	% Triggering reflex
1	1	3	28.02(0.16)	27.50(0.73)	29.57(0.91)	28.96(0.76)	N/A
	8	4	28.05(0.19)	28.46(0.16)	30.69(0.34)	28.54(0.56)	100%
	16	4	28.93(0.58)	29.97(0.59)	31.62(0.72)	28.71(0.59)	100%
	24	12	28.45(0.69)	29.29(1.04)	31.72(0.41)	28.9(1.33)	100%
	32	4	28.44(0.47)	29.7(0.71)	31.55(0.25)	30.02(0.32)	100%
	48	4	28.52(0.5)	30.04(1.02)	32.08(0.84)	29.17(0.81)	100%
2	1	3	26.03(0.41)	26.09(0.21)	28.32(0.29)	28.34(2.39)	N/A
	8	3	28.39(0.24)	27.74(1.74)	30.25(1.42)	28.25(1.58)	100%
	16	3	25.96(0.97)	28.64(0.75)	31.43(0.08)	29.63(0.69)	100%
	24	12	27.67(0.63)	29.03(0.97)	32.19(0.4)	28.48(1.35)	100%
	32	3	28.28(0.8)	29.6(0.39)	33.13(1)	29.2(0.09)	100%
	48	3	26.96(0.67)	30.03(0.8)	33.45(0.2)	30.13(0.64)	100%
3	1	3	27.93(0.06)	31.13(0.58)	28.92(1.62)	27.95(2.31)	N/A
	8	3	29.05(0.22)	29(0.52)	30.53(0.3)	28.89(0.16)	100%
	24	9	28.82(1.29)	30.16(0.85)	31.9(0.61)	30.25(0.88)	100%
	32	3	31.06(0.4)	31.49(1.34)	33.74(0.15)	32.58(0.21)	100%
	48	3	29.61(0.22)	31.03(0.89)	33.85(0.54)	31.02(1.35)	100%
4	1	3	25.13(0.03)	26.89(0.15)	29.35(0.15)	27.71(0.24)	N/A
	8	3	26.93(0.06)	28.84(0.15)	30.81(0.07)	29.2(0.18)	100%
	16	3	25.19(0.02)	28.41(0.08)	30.93(0.07)	29.48(0.45)	100%
	24	3	24.77(0.06)	29.1(0.17)	31.17(0.26)	29.56(0.14)	100%
5	1	3	25.88(0.23)	28.39(0.1)	31.25(0.21)	29.75(0.21)	N/A
	8	3	27.13(0.13)	29.35(0.39)	33.19(0.39)	31.46(0.59)	100%
	16	3	25.72(0.06)	29.9(0.11)	32.53(0.32)	31.1(0.32)	100%
	24	3	26.87(0.07)	30.11(0.46)	33.16(0.6)	31.55(1.22)	100%
6	1	3	25.86(1)	31.54(0.58)	31.15(0.14)	31.62(1.03)	N/A
	8	3	27.91(0.11)	31.61(0.67)	33.36(0.18)	32.45(0.33)	100%
	24	9	28.55(1.35)	31.9(0.76)	33.91(0.81)	32.26(1)	100%
7	1	3	26.65(0.9)	26.32(0.24)	26.44(0.04)	26.36(0.33)	N/A
	24	6	25.41(0.42)	28.09(0.32)	29.63(0.19)	27.76(0.28)	100%
8	1	3	28.97(0.1)	29.77(0.01)	29.05(0.29)	28.59(0.63)	N/A
	24	6	28.85(1.18)	31.96(0.29)	32.85(0.35)	29.42(1.14)	100%
9	1	3	31.05(1.46)	30.25(1.18)	29.55(0.44)	30.33(0.62)	N/A
	24	6	27.59(0.54)	31.11(0.34)	31.5(0.2)	29.47(0.14)	100%

We also compared the detection of SARS-CoV-2 in saliva using the SalivaClear࣪ method and in the reference testing nasopharyngeal swab modality in 20 positive and 100 negative samples (Table 4). The saliva and swab samples were collected concurrently. The swab specimens were sent to a tertiary laboratory to be tested using an FDA EUA high sensitivity testing platform for SARS-CoV-2 detection, Roche Cobas 6800. There was 100% agreement between the results of saliva versus nasopharyngeal swab diagnostic testing (kappa coefficient = 1, perfect agreement). The Wilcoxon signed-rank test showed that the median Ct value for the common viral target (ORF1ab) of the nasopharyngeal swab was significantly different from the median Ct value of the saliva test (*Z* = −3·267, *p* = 0·001). Indeed, the median Ct value for ORF1ab of the nasopharyngeal swab was 30.35 and the median Ct value of the concurrently collected saliva specimens was only 21.04. These results may indicate any of the following possibilities: (1) higher viral load in the saliva, (2) higher sensitivity of the saliva test, (3) better viral recovery and/or stability using saliva collection.

**Table 4:** Comparison of detection of SARS-CoV-2 in saliva using the TaqPath and in concurrently collected nasopharyngeal swab using an FDA EUA high sensitivity testing platform, Roche COBAS 6800.

Sample Number	Nasopharyngeal Swab(Roche cobas 6800)				Saliva(SalivaClear)					
	ORF1ab	E gene	Internal Control	Result	MS2	N gene	ORF1ab	S gene	RP	Result
1	29·24	30·12	33·02	POS	25·66	20·08	19·59	20·13	22·16	POS
2	15·39	15·76	33·43	POS	25·37	20·51	19·81	20·68	23·44	POS
3	18·05	18·29	33·43	POS	25·66	19·97	19·65	20·46	23·04	POS
4	30·58	31·6	32·95	POS	25·54	20·64	20·29	20·9	22·63	POS
5	18·02	18·31	33·48	POS	25·34	19·87	19·54	19·98	22·29	POS
6	30·11	31·14	32·98	POS	25·38	19·58	19·34	19·79	22·23	POS
7	15·49	15·81	33·58	POS	26·02	19·45	19·52	20·10	22·41	POS
8	34·8	37·43	33·04	POS	25·59	20·17	19·89	20·38	22·57	POS
9	24·6	25·09	32·75	POS	25·45	19·69	19·17	19·82	22·09	POS
10	24·99	25·46	32·96	POS	25·51	19·99	19·63	20·05	22·25	POS
11	33·98	35·83	33·17	POS	25·19	22·47	21·97	22·3	26·19	POS
12	23·3	23·73	33·12	POS	24·95	22·16	21·78	22·22	26·61	POS
13	35·71	37·09	33·3	POS	25·43	22·78	22·58	23·04	27·41	POS
14	27·85	28·33	32·85	POS	25·07	21·89	21·82	21·84	26·80	POS
15	33·66	34·95	33·25	POS	28·28	28·6	29·15	ND	19·72	POS
16	32·59	34·74	34·44	POS	27·61	27·59	28·08	ND	19·46	POS
17	34·73	37·93	33·41	POS	26·34	28·34	28·54	ND	20·28	POS
18	33·2	34·77	33·22	POS	28·57	28·61	29·53	ND	19·91	POS
19	32·43	34·75	33·11	POS	26·40	27·68	28·00	ND	20·80	POS
20	31·78	33·83	35·54	POS	26·73	28·43	28·31	ND	19·77	POS
21–120	ND	ND	33·60	NEG	26·64	ND	ND	ND	24·07	NEG

POS – positive; ND – not detected; NEG – negative.

**Fig. 3:**
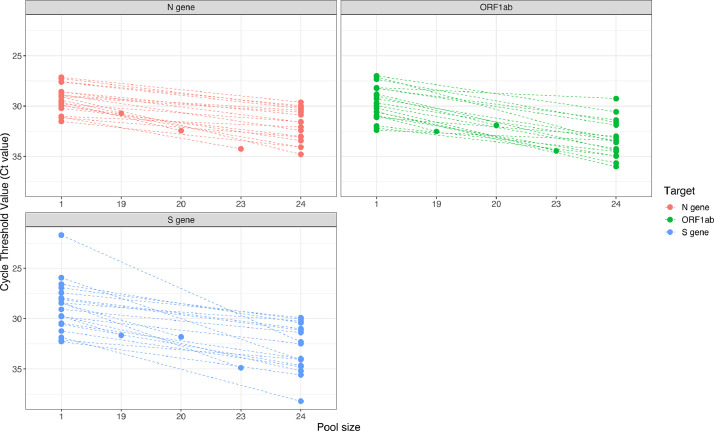
Ct value comparisons between pooled and individual samples. Twenty randomly selected positive samples were analyzed and Ct values compared between a pool of 24 samples and a single sample. The dashed lines connect the dots that indicate the Ct values of individual samples (higher) to the corresponding pools (lower).

From the sample pools obtained through SalivaClear࣪ testing, we randomly selected 20 positive pools and compared the Ct values of the pool to the corresponding positive individual sample in that pool. There was a 100% agreement in the detection of viral targets between the pools and individual samples (Fig. 3; Supplementary Table 3). The mean shift in Ct values between the pooled and individual sample was 2·1, 3·0 and 3·2 for the three targets (*N* gene, *S* gene, ORF1ab gene), respectively. Approximately 2% of all negative pools were deconvoluted to check if some positive individual samples may be missed by pooling. All individual samples also tested negative (data not shown).

During the 20-week period from August 27, 2020 to January 13, 2021, 237,164 specimens condensed into 10,719 pools were collected from K-12 students and faculty and staff at 93 schools (Supplementary Table 4). An additional 16,242 specimens condensed into 754 pools were collected from 19 universities (Supplementary Table 4). The number of pools collected from each of the 112 institutions ranged from 1 to 92 pools (mean=13, median=7) per school on each day. Each pool contained two to 24 (mean=21, median=23) individual saliva samples. Of the 114,73 pools, 542 pools were positive (4·7% pool positivity rate), with zero to 56 (mean=4·9, median=2) positive pools per institution. A total of 855 positive individual samples (0·3% individual positivity rate) were detected using this method. Each positive pool had 1–10 (mean=1·6, median=1) samples that tested positive on deconvolution testing. Table 5 shows the summary of these findings.

**Table 5:** Summary of data collected from K-12 schools and universities.

Summary	K-12	University	All Institutions
Total number of schools	93	19	112
Total number of pools	10,719	754	11,473
Total number of individual samples	237,164	16,242	253,406
Total number of positive pools	452	90	542
Mean (SD) of% positive pools per institution	0·38(0·63)	0·85(0·64)	0·47(0·65)
Median of% positive pools per institution	0·16	0·77	0·22
Total number of positive individuals	665	190	855
Mean (SD) of% positive individuals per institution	0·04(0·12)	0·11(0·17)	0·05(0·13)
Median of% positive individuals per institution	0·01	0·05	0·02

**Fig. 4:**
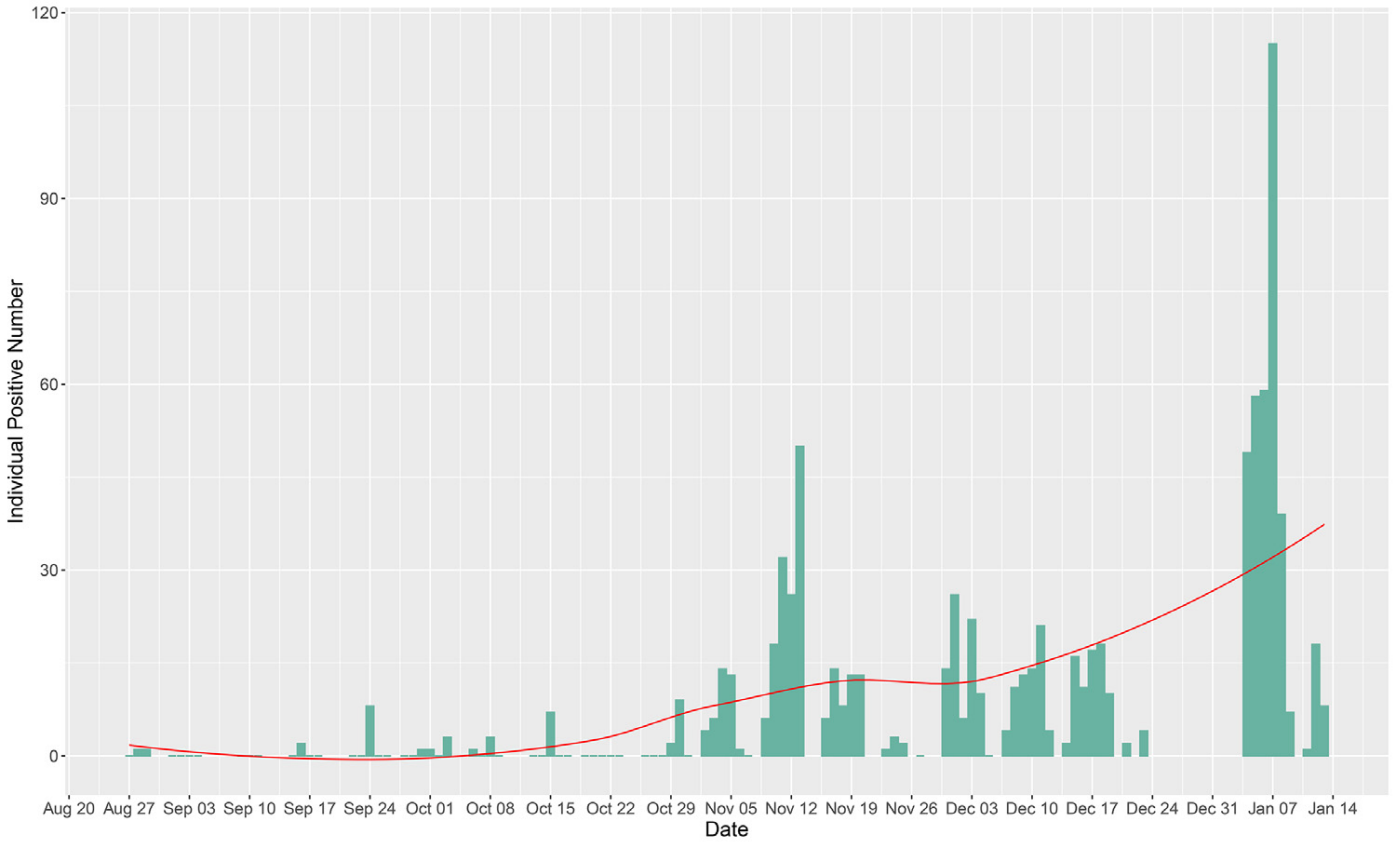
Trend of saliva-based pooled testing positivity in 109 K-12 schools and universities for a five-month period. Peaks of positive cases (individual samples) were observed in dates following October 31, 2020 (Halloween) and December 31st, 2020 (New Year's Eve). The green bar lines indicate the number of positive individual samples, and the red curve is an estimation of the trend. (For interpretation of the references to color in this figure legend, the reader is referred to the web version of this article.)

In analyzing the trend of positivity among the pools (Fig. 4), observable peaks were seen on days following long weekends and holidays. The highest number of positive pools was observed 5 days after New Year's Eve (January 7, 2021; 39 positive pools out of 224, with 115 positive individuals out of 4791). The second highest number of positive pools was recorded on November 11 (29 positive pools out of 296; 32 positive individuals out of 6731) and the second highest number of positive individual samples was observed on November 13 (25 positive pools out of 234; 50 positive individuals out of 5181), 11–13 days after October 31st, 2020 (Halloween). Of note, the SalivaClear࣪ method was able to identify 10 positive (out of 16) pools for one school campus on November 13, corresponding to 26 positive individuals who attended an off-campus Halloween celebration. The saliva pooling method also identified 10 positive (out of 64) pools from another school on November 10, yet all positive individuals from these pools had reported separate exposures. The third highest number of positive pools was on December 1 and December 3 (21 positive pools each; 26 and 22 positive individuals, respectively), 5–7 days after November 26, 2020 (Thanksgiving). Supplementary Tables 5 and 6 show the outlier detection analyses in which November 13 and January 4, 6, and 7 were all determined to be outliers.

**Fig. 5:**
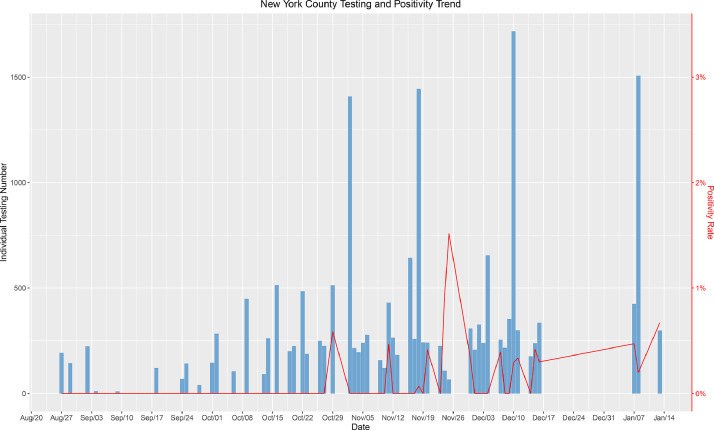
A graph of the aggregated positivity rate and aggregated number of tests done between the 3 schools we have tested most frequently in New York County, New York. The bar lines indicate the total number of tests done, and the red line indicates the percentage of positive test results from the total tests performed on a given date. (For interpretation of the references to color in this figure legend, the reader is referred to the web version of this article.)

**Fig. 6:**
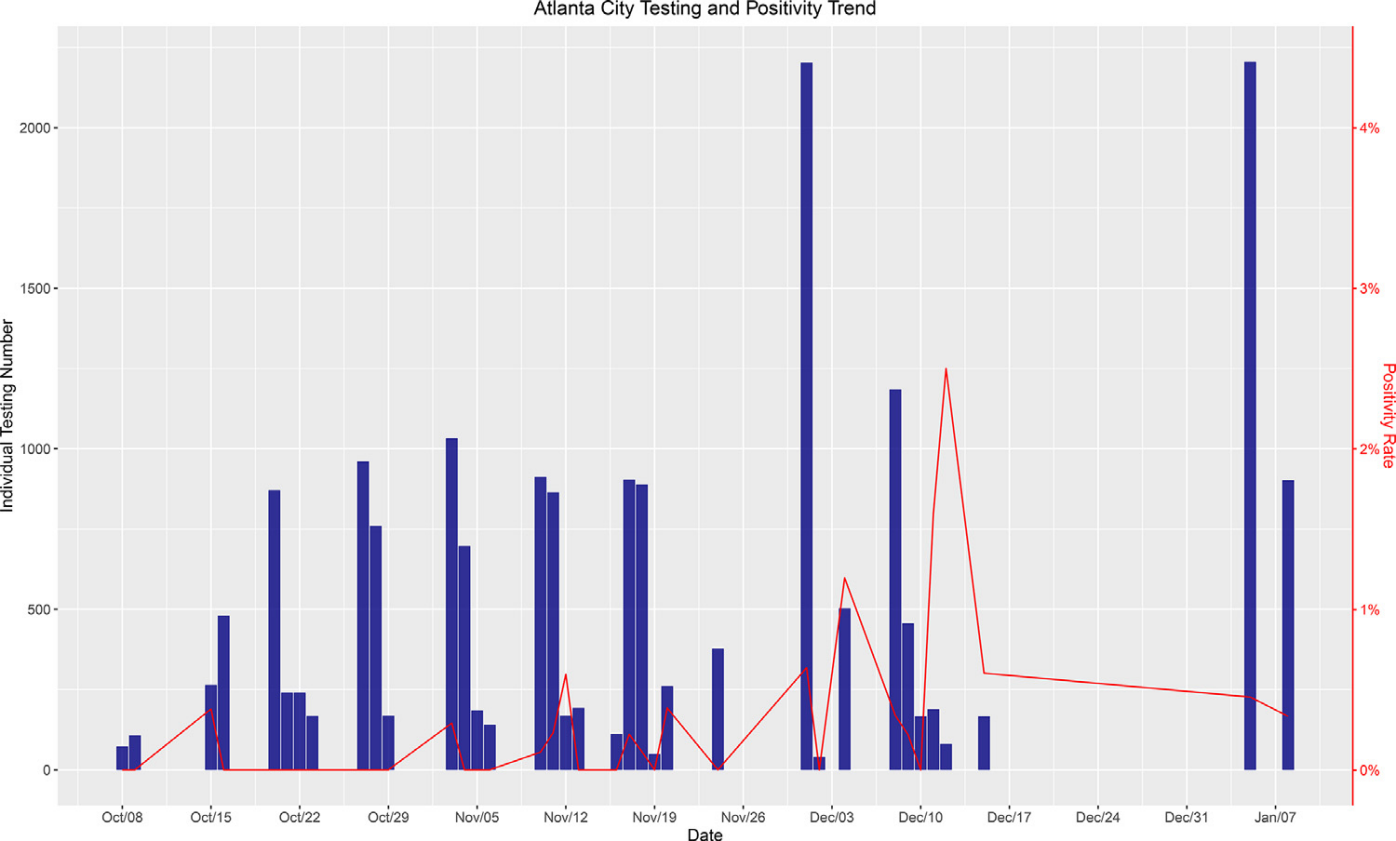
A graph of the aggregated positivity rate and aggregated number of tests performed between the 3 schools we have tested most frequently in Atlanta, Georgia. The bar lines indicate the number of total tests done, and the red line indicates the percentage of positive test results obtained. (For interpretation of the references to color in this figure legend, the reader is referred to the web version of this article.)

Next, we compared the positivity of our test population to that of the community. We selected 3 New York county schools and universities that we tested most frequently. Fig. 5 demonstrates the number of tests and the positivity rate in these three schools. With the exception of 2 low volume testing days (<500 samples), our positivity rate remained between 0 to approximately 0.5%, which was dramatically lower than the New York County reported positivity rate of 1–4% between November 1 and January 13 (https://forward.ny.gov/percentage-positive-results-county-dashboard). Between August 27 and November 1, the positivity rates were approximately the same in the schools we tested and in the general population of New York county (0.75%). After November 1, the positivity rate within the schools remained consistently lower (50−87%) than the county positivity rate after November 1. A similar trend was observed for a school in Fulton County, which is mostly comprised by the city of Atlanta, GA (Fig. 6). With the exception of two low volume testing dates, the positivity rate within the school ranged from 0 and approximately 1% while Fulton County reported positivity rates ranging between 4.5 and 15% during the same time period from August 27 to January 9 (https://dph.georgia.gov/covid-19-daily-status-report). These results from two unique geographies reveal that the actual prevalence of infection was consistently lower in schools undergoing surveillance testing than the general community, strongly suggesting that viral transmission and infection were less likely to occur within school premises.

On average, the results of pooled testing were available and reported to the institutions within 6–12 h from specimen accessioning and within 18–30 h from specimen collection, accounting largely for overnight shipping time. In the event of SARS-CoV-2 detection within a pool, individual results were reported within 6–8 h following release of a pooled result. Therefore, on average, an individual diagnostic result, following two rounds of testing, was provided within 24–38 h from specimen collection, with travel time playing the largest factor in the turn-around-time. The cost of the initial pooled testing was on average $10 (range: $8–12) per individual for all participating schools. Reflex testing included additional charges, with the overall pooled to individual test averaging $12.5 (range: $10–15) per person. This included all testing supplies and reagents, logistics, and personnel as well as diagnostic reporting, and allowed for over 12 districts to participate in the current study. The average cost of the nasopharyngeal swab RT-PCR testing was $127 (range: $20–850) per person [21]. Most of these swab tests (51%) were priced between $100 and 199, and nearly one in five (19%) were priced above $200. For an individual participating in the pooled saliva testing, the maximum cost of testing (average of $22.5, including possible reflex testing for positive pools) was $104.5 less than the average cost of a nasopharyngeal swab test. For a school with a student population of 500, this difference will amount to a $52,250 cost-minimization per testing cycle. Weekly cycles using nasopharyngeal swab would cost approximately $63,500 per week, $254,000 in a month and $2,286,000 in a school year. Weekly cycles with saliva pooled testing cost $11,250 per testing cycle, $45,000 in a month and $405,000 in a school year. This amounts to a total cost-minimization of $1,881,000 per school year.

Surveillance testing was a factor in the early detection of asymptomatic infection and minimization of an outbreak risk. According to our data, a 0.3% positivity rate was observed after testing 253,406 samples. This amounts to 855 individuals identified early before a significant outbreak was observed. Although it would be difficult to estimate cost averted due to variability of positivity rate among asymptomatic populations, we estimated that these 855 individuals could potentially spread infection to 2.4 persons per day in a school setting [22]. In the absence of an early detection strategy (surveillance), this would lead to 2052 infected persons due to asymptomatic transmission. Out of these 2052 individuals, up to 25% (or 513 persons) may be symptomatic [23]. These persons would have required out-patient management at minimum, costing an average of $500–1000 for out-patient care, according to data released by Blue Cross Blue Shield [24]. Therefore, the costs averted for mild to moderate cases can be conservatively estimated to be between $256,500 and 513,000. Furthermore, nearly 5% of symptomatic individuals (under 20 years old) have presented with severe to critical disease [25,26], Thus nearly 26 individuals would have required hospitalization according to a FAIR Health Study, with the median cost of hospitalization ranging from a low of $34,662 for the 23–30 age group to a high of $45,683 for the 51–60 age group [24]. For those under 20 years of age, the average hospitalization cost was estimated at $68,261 and $77,323 for those over 60 years of age. Therefore, the cost averted for severe to critical cases was conservatively estimated to be $901,212 for our test population. These were the most conservative estimates of the cost averted by combining frequent surveillance testing (weekly) with prompt isolation/quarantine procedures in school and university setting. These estimates did not consider the worst-case scenario, where infection to older individuals within the school (teachers, principal) and outside of the school setting (parents, grandparents) would most likely have led to worse clinical outcome due to COVID-19.

## 4 Discussion

The closure of schools has been one of the most adverse impacts of COVID-19 among children [27]. Other than negatively affecting learning aptitude especially in younger children, school closures have been linked to several psychosocial problems [28]. The full negative impact of school closures due to COVID-19 throughout the world will remain unknown for years to come; however, the effects on mental health, nutrition, increasing educational gaps and socioeconomical inequality have already become apparent [27]. In the 5th largest school district in the nation, schools were forced to reopen despite continuously high positivity rates due to a surge student suicide [29], demonstrating how important schools are for meeting both academic and nonacademic needs of our nation's children.

To meet the COVID-19 testing demands needed for schools to reopen and remain open by use of mitigation strategies including testing, we sought to create an organization-based pooling strategy, whereby schools could enroll in surveillance testing to monitor groups of people through pooled testing. By using organization-based pooling, the program avoided random pooling of samples. Instead, our approach pooled groups of known contacts from school where students, faculty and staff regularly interacted utilizing a similar protocol. By this approach, a positive case impacted the entire pooled population, and the entire pooled population was treated as an infected cohort until further individual testing was completed and reported through a healthcare provider.

Here, we report on testing of over 250,000 COVID-19 RT-PCR specimens from students, faculty and staff from 93 K-12 schools and 18 universities during a 20-week period during this pandemic. The pooled testing was facilitated by trained collection specialists and utilized local logistics companies, pre-barcoded tubes, and automation to expedite sample processing. We expect this passive drool method minimized the potential aerosolization of infectious agents as seen in involuntary coughing and sneezing encountered with the swab methods (nasopharyngeal, oropharyngeal, nasal). These factors enabled the collection and assessment of up to 25,000 tests per day, highlighting the potential to scale such a method.

Importantly, we found that SalivaClear࣪ has a similar sensitivity to the molecular assay of individual samples, in terms of both qualitative (100% agreement of results on both pooled and individual samples) and quantitative (comparable Ct values between pooled and individual samples) measures. Without sacrificing the reliability of the molecular assay, pooling of samples had substantially reduced the costs associated with PCR testing. In order to broadly test communities and schools, lower pricing and pooled approaches in particular allow for a “multiplier effect” that can provide significant economies of scale, potentially decreasing the costs even further at larger scale.

Another low-cost surveillance method that has recently gained popularity is waste-water surveillance, used to measure excreted viral particles in sewage. Wastewater surveillance is a less expensive and less invasive method in assessing the epidemiological trends of COVID-19. However, recent studies have shown that detection of viral RNA in wastewater sludge did not provide a significant early warning and lacked predictive power before an outbreak [30]. Sludge viral levels were found to mirror the trend of hospitalizations and positive cases. The exact sensitivity of this method is unknown, and a study found viral RNA copies ranging from 1.7 × 10^3^ to 4.6 × 10^5^ per ml of the primary sludge [30]. This method also does not allow rapid identification of the infected individual. The waste-water method will only identify virus shedding in an establishment but will not be able to identify the source unless another specimen is collected from all individuals. This will lead to delays and subsequently more cost for this method to have actionable results. The major benefit of our saliva pooled testing is that it allows for reflex deconvolution to identify the individual with a positive result using the same specimen. The saliva testing stands as a more precise measurement of an individual's infectiousness as the virus can effectively be transmitted through the saliva and respiratory droplets [31]. The virus is inactivated rapidly in the gastrointestinal tract fluid and may still be excreted through the feces even during the non-infectious period [32].

The rapid turnaround time of pooled results allowed schools to assess transmission and adjust prevention protocols in a timely manner. As a result of sampling at the same time, coupled with grouping by work area, grade, or section, pool results helped institution administrators to determine if transmission occurred, make data driven decisions, and adjust and improve safety protocols. The surveillance program is just one component of the established protocols essential for preventing transmission of COVID-19 within the school premises and limiting outbreaks. In one instance, there was strong evidence of in-school transmission of the virus as determined by the surveillance and subsequent individual sample testing and contact tracing within the main office. This led to a review of heating, ventilating and air-conditioning (HVAC) systems as well as air circulation mapping throughout the workstations. Surprisingly, although plexiglass dividers were installed to prevent viral transmission, the air circulation testing using smoke devices revealed that plexiglass dividers, when coupled with side panels, significantly impeded air circulation leading to increased viral transmission.

Several studies have shown reliable detection of SARS-CoV-2 RNA in saliva samples [33], [34], [35], [36], [37], [38]. In a few of them, saliva samples had superior sensitivity and stability compared to the nasopharyngeal swab [33,37,39,40]. However, there were studies that found lower detection of the virus in saliva when compared to concurrently collected nasopharyngeal swab samples. One meta-analysis of 49 studies found that nasopharyngeal/oropharyngeal swabs had 5% higher sensitivity (92.2%) as compared to saliva (86.7%) [41]. Another study compared the sensitivity of SARS-CoV-2 detection in saliva and concurrently collected nasopharyngeal swab [42]. The positive agreement of saliva and swab was only 81.1% (95% CI, 65.8–90.5%), but increased to 90.0% for high viral load samples. Although the sensitivity of detection in saliva was lower than that of the swabs, the authors still concluded that saliva was adequate for detection of individuals with higher viral loads in an asymptomatic screening program as it does not require viral transport medium and may help improve screening compliance. Despite the variable perspective on the reliability of saliva in SARS-CoV-2 diagnosis, there are several advantages that make saliva more attractive as a surveillance specimen. Saliva is stable even when collected in a dry, sterile container; [40,43] easy to collect and may improve compliance to frequent testing; [42] can be self-collected under minimal supervision, thereby precluding the unnecessary risk of exposure to healthcare workers; and carries lower risk of adverse outcomes compared to swabs [40]. More importantly for a pandemic event, nasopharyngeal swabs were limited by finite supply chains and restricted distribution. Similar to published data [40,43], we demonstrated adequate stability of saliva samples when collected in dry, sterile collection containers, thereby expanding the options for COVID-19 specimen collection device (Supplementary Table 7).

The detection of asymptomatic infected SARS-CoV-2 carriers is the main purpose of performing surveillance testing. A study of 360 individual saliva samples pooled into 30 sets (pool size of 12 samples) observed higher efficiency of detecting 5% asymptomatic infected individuals than the swabbing method and 82% of PCR reagents was spared [14]. As we increased our pool size to 24, the estimated average conservation of resources was nearly 95%. On the day when we had the highest number of positive pools, which was on January 7, 2021, we had to reflex 17.1% of pools (39 out of 224) and were able to detect a total of 115 cases out of 4791 (2.4%). To do so, we conducted 1060 tests to cover all 4791 individuals, which was only 22% of the total tests required to perform individual testing. Other studies have explored pooling of saliva with pools of 5 to 6 samples, and these studies observed approximately 90– 95% sensitivity when compared to individual testing [42,43]. Another study compared the sensitivity of pooling based on the number of pooled samples [12]. Similar to our study, pools of 5, 10 and 20 samples were prepared prior to RNA extraction, and there was an estimated reduction in sensitivity (7.41%, 11.11% and 14.81%, respectively) for each of the pool sizes. Although an expected decrease in sensitivity was observed from pooling, changing some parameters of testing, such as increasing the extraction volume and Ct or Ct value threshold, would improve sensitivity comparable to undiluted or unpooled levels [4,5,14,16]. We presented a method where the extraction volume was increased from 100 to 200 µl and the Ct value threshold to reflex for individual testing was increased from 37 to 40. In addition, the RT-PCR was performed in triplicate reactions providing a total of 9 potential amplifications that would trigger reflex of the pool. These simple adjustments allowed us to maintain sensitivity similar to individual testing, while at the same time conserving resources even in a prevalence of greater than 5%. These protocol adjustments were also demonstrated by a study conducted in Thailand [44], where they performed individual RNA extraction of 200 specimens and pooled 10 µl of the RNA extracts into pools of 5 and 10 samples. This study utilized a Ct value of at most 45 for a positive pool and detected positive individual samples in all their pools.

Here we describe the pooled method of SalivaClear࣪ that uses a large pool size that has not previously been described in the literature, enabling a greater “multiplier” effect to increase testing capacity across the country. With testing result delays across the country, primarily due to reagent and capacity shortages, pooling enables conservation of resources while scaling capacity and still being able to provide individualized results when required. Our data shows that pooled saliva testing performed with the highest efficiency in terms of timeliness and cost was one of the crucial solutions supporting school organizations to safely conduct in-person classes amidst the pandemic crisis.

One limitation of this study is that the applicability of the methodology described here requires both technical and logistical methods that cannot be easily reproduced in every lab. However, concepts of pooling, strategies around reducing costs for schools, and the unique value of providing testing for schools as it relates to community viral spread described here, remain broadly applicable to the K-12 testing strategies that are required around the country. Additional limitations include the need for laboratory-based testing as in theory this concept can be broadly applied to point of care concepts as well. However, as with any testing strategy, scalability is important. The ability to rapidly scale SalivaClear࣪ using existing lab infrastructure makes it broadly appealing to established clinical laboratories seeking to increase testing capacity and efficiency. While the non-standardized methods of school openings across the country are a limiting factor when evaluating the data for the exact role SalivaClear࣪ played for each school, it is important to highlight that all schools utilizing this method were able to remain open.

In order to provide enough testing capacity to safely open schools and provide testing for at-risk communities, pooled concepts must be further evaluated and considered. As vaccination against COVID-19 may remain unavailable for children under 16 years of age for months to come, pooled testing must be considered a fundamental component of the safe reopening of schools while minimizing transmission among students, administration and faculty.

## Declaration of Competing Interest

None.

## Appendix Supplementary materials

Image, application 1

Image, application 2

Image, application 3

Image, application 4

Image, application 5

Image, application 6

Image, application 7

Image, application 8
