# Supplementary material for: Implementation of a pooled surveillance testing program for asymptomatic SARS-CoV-2 infections in K-12 schools and universities
Source: eClinicalMedicine. 2021 Jul 17;38:101028. doi: 10.1016/j.eclinm.2021.101028 (PMC8286123; doi:10.1016/j.eclinm.2021.101028)
Supplement: Supplementary file 1 [file mmc1.pdf]

Supplementary Table 1. Summary of LOD preliminary range finding in individual specimen using known concentrations of ATCC VR-1986HK whole inactivated virus spiked into individual negative saliva. The stock concentration provided by ATCC was 4.2 x10^5 GCE/µL. For each RNA extraction, 100µL of sample was used.

Viral Conc	Replicate	Mean Ct (SD,N=3)					Result	% Pos
		Orf1ab	N	S	MS2	RP		
5000 GCE/ml (25 GCE/rxn)	1	28.6(0.39)	27.53(1.16)	27.48(0.66)	33.78(NA)	24.69(0.07)	Positive	100%
	2	28.84(0.44)	26.59(0.75)	27.3(0.44)	ND	24.83(0.09)	Positive	
	3	28.83(0.22)	28.01(0.66)	27.02(0.76)	ND	25.1(0.06)	Positive	
2500 GCE/ml (12.5 GCE/rxn)	1	29.22(0.64)	25.99(2.03)	28.69(NA)	28.21(3.2)	24.63(0.04)	Positive	100%
	2	29.02(0.48)	26.73(1.12)	28.12(0.41)	29.57(0.51)	24.43(0.03)	Positive	
	3	29.48(0.28)	28.34(0.81)	28.22(0.74)	31.62(1.21)	24.62(0.08)	Positive	
1250 GCE/ml (6.3 GCE/rxn)	1	30.66(0.43)	28.13(NA)	29.52(0.26)	ND	24.56(0.03)	Positive	100%
	2	32.11(NA)	28.8(2.89)	30.97(NA)	ND	24.44(0.31)	Positive	
	3	30.88(1.29)	31.4(0.27)	28.99(0.07)	ND	24.69(0.05)	Positive	
625 GCE/ml (3.1 GCE/rxn)	1	30.56(0.07)	30.94(0.58)	30.6(NA)	ND	24.84(0.07)	Positive	100%
	2	29.07(0.25)	29.74(NA)	ND	32.67(1.97)	24.92(0.06)	Positive	
	3	30.49(1.32)	29.6(0.18)	28.48(NA)	31.48(0.38)	24.45(0.5)	Positive	
313 GCE/ml (1.6 GCE/rxn)	1	33.04(1.02)	28.27(NA)	ND	31.11(2.84)	24.33(0.08)	Positive	100%
	2	31.84(NA)	ND	ND	31.8(NA)	24.43(0.09)	Inconclusive	
	3	30.5(1.16)	30.49(NA)	28.61(2.27)	32.08(NA)	24.37(0.08)	Positive	
156 GCE/ml (0.8 GCE/rxn)	1	32.27(NA)	ND	ND	33.33(NA)	24.41(0.07)	Inconclusive	66.67%
	2	ND	ND	ND	34.43(NA)	24.55(0.13)	Negative	
	3	33.56(NA)	ND	ND	34.66(NA)	24.55(0.1)	Inconclusive	
78 GCE/ml (0.4 GCE/rxn)	1	ND	ND	ND	32.83(NA)	24.72(0.08)	Negative	66.67%
	2	32.14(NA)	ND	ND	31.39(0.11)	24.77(0.07)	Inconclusive	
	3	32.62(NA)	ND	ND	28.02(6.64)	24.49(0.06)	Inconclusive	
39 GCE/ml (0.2 GCE/rxn)	1	ND	ND	ND	ND	24.55(0.06)	Negative	0%
	2	ND	ND	ND	33.75(NA)	24.57(0.05)	Negative	
	3	ND	ND	ND	ND	24.56(0.08)	Negative	
20 GCE/ml (0.1 GCE/rxn)	1	ND	ND	ND	34.64(NA)	24.68(0.07)	Negative	0%
	2	ND	ND	ND	ND	24.72(0.17)	Negative	
	3	ND	ND	ND	ND	24.63(0.45)	Negative	
10 GCE/ml (0.05 GCE/rxn)	1	ND	ND	ND	31.04(0.23)	24.62(0.04)	Negative	0%
	2	ND	ND	ND	28.44(1.72)	24.81(0.18)	Negative	
	3	ND	ND	ND	ND	24.71(0.46)	Negative	
5 GCE/ml (0.025 GCE/rxn)	1	ND	ND	ND	ND	24.55(0.1)	Negative	0%
	2	ND	ND	ND	ND	24.82(0.11)	Negative	
	3	ND	ND	ND	ND	24.88(0.11)	Negative	

N = number of RNA extractions.
