# Supplementary material for: Implementation of a pooled surveillance testing program for asymptomatic SARS-CoV-2 infections in K-12 schools and universities
Source: eClinicalMedicine. 2021 Jul 17;38:101028. doi: 10.1016/j.eclinm.2021.101028 (PMC8286123; doi:10.1016/j.eclinm.2021.101028)
Supplement: Supplementary file 2 [file mmc2.pdf]

Supplementary Table 2. Summary of LOD preliminary range finding in pooled specimens using known concentrations of ATCC VR-1986HK whole inactivated virus spiked into individual negative saliva. The stock concentration provided by ATCC was $4.2x10^5$ GCE/µL. For each RNA extraction, 200µL of sample was used.

**Viral Conc**	**Pool size**	**N**	**Mean Ct (SD; N=3)**					Result	% Triggering Reflex
			**Orf1ab**	**N gene**	**S gene**	**MS2**	**RP**		
1250 GCE/ml (6.3 GCE/rxn)	1	1	29.96(0.3)	28.37(0.34)	24.94(4.28)	28.67(1.06)	23.29(0.45)	Positive	100%
		2	29.99(0.43)	28.51(1.44)	25.75(5.33)	28.11(0.47)	23.04(0.04)	Positive	
		3	30.67(1.01)	28.86(2)	26.36(2.34)	28.27(0.28)	22.88(0.16)	Positive	
	8	1	32.83(0.62)	30.18(0.31)	30.25(0.75)	27.9(0.5)	23.19(0.08)	Positive	100%
		2	31.97(0.64)	30.4(0.1)	27.73(1.87)	28.24(0.41)	23.32(0.11)	Positive	
		3	32.36(1.18)	30.11(2.73)	25.88(4.4)	28.51(0.4)	23.23(0.07)	Positive	
	16	1	33.37(0.6)	31.89(1.04)	33.27(NA)	27.98(0.57)	23.02(0.07)	Positive	100%
		2	32.28(NA)	ND	ND	28.4(0.93)	23.12(0.02)	Inconclusive	
		3	ND	31.38(NA)	28.46(NA)	28.9(0.59)	23.19(0.14)	Positive	
	24	1	32.65(1.13)	31.87(NA)	31.05(NA)	28.16(0.92)	23(0.04)	Positive	95.83%
		2	33.74(0.03)	30.8(NA)	ND	28.54(0.86)	23.08(0.04)	Positive	
		3	33.76(0.43)	ND	30.01(0.9)	29.22(0.37)	23.03(0.11)	Positive	
		4	31.29(NA)	30.5(NA)	ND	27.23(0.97)	22.72(0.05)	Positive	
		5	32.58(0.12)	32.47(NA)	ND	27.04(0.7)	22.79(0.22)	Positive	
		6	32.96(0.6)	ND	24.91(NA)	27.34(1.11)	22.83(0.07)	Positive	
		7	33.76(NA)	ND	ND	27.63(0.3)	22.95(0.23)	Inconclusive	
		8	30.88(NA)	ND	ND	27.79(0.77)	23.17(0.13)	Inconclusive	
		9	33.62(NA)	32.82(NA)	32.23(0.12)	27.83(0.37)	22.7(0.15)	Positive	
		10	33.52(NA)	31.03(NA)	ND	28.24(0.52)	23.3(0.06)	Positive	
		11	32.73(NA)	ND	27.8(NA)	27.89(0.26)	22.98(0.12)	Positive	
		12	33.31(1.73)	30.85(NA)	30.5(NA)	28.03(0.63)	23.1(0.03)	Positive	
		13	32.36(NA)	31.92(NA)	ND	27.87(0.44)	22.96(0.05)	Positive	
		14	32.53(NA)	31.87(0.29)	30.92(NA)	28.24(0.09)	23.09(0.13)	Positive	
		15	32.6(0.37)	31.34(1.44)	27.65(3.36)	27.81(0.22)	22.8(0.18)	Positive	
		16	32.54(NA)	ND	ND	28.37(0.57)	23.05(0.02)	Inconclusive	
		17	ND	ND	29.7(NA)	27.94(0.3)	23.14(0.1)	Inconclusive	
		18	ND	ND	ND	28.21(0.67)	23.05(0.03)	Negative	
		19	33.07(NA)	ND	30.85(1.38)	27.91(0.21)	23.22(0.11)	Positive	
		20	32.86(NA)	ND	32.46(NA)	27.79(0.65)	23.31(0.14)	Positive	
	36	1	34.12(NA)	29.38(NA)	ND	28.36(0.56)	23.02(0.05)	Positive	100%
		2	33.87(NA)	30.69(2.08)	25.13(NA)	28.6(0.73)	23.06(0.08)	Positive	
		3	33.56(NA)	25.9(0.05)	ND	28.13(1.09)	22.95(0.04)	Positive	
	48	1	33.78(NA)	31.32(NA)	ND	28.05(0.76)	23.1(0.04)	Positive	66.67%
		2	ND	32.12(NA)	31.42(NA)	28.41(0.49)	23.35(0.05)	Positive	
		3	ND	ND	ND	27.86(1.17)	23.09(0.05)	Negative	

625 GCE/ml (3.1 GCE/rxn	1	1	31.85(1.09)	30.39(0.78)	26.45(4.58)	28.14(0.33)	22.83(0.13)	Positive	100%
		2	30.96(0.69)	29.31(0.73)	26.05(6.23)	28.25(0.44)	22.99(0.05)	Positive	
		3	31.22(0.89)	29.51(0.46)	27.27(2.12)	28.49(0.57)	23.02(0.04)	Positive	
	8	1	33.47(NA)	ND	ND	28.55(0.58)	23.16(0.05)	Inconclusive	100%
		2	33.97(0.43)	27.84(4.59)	28.67(NA)	28.27(0.91)	23.07(0.04)	Positive	
		3	33.78(0.78)	32.21(0.03)	26.21(NA)	28.53(0.57)	23.41(0.08)	Positive	
	16	1	33.5(1.48)	30.87(NA)	25.5(NA)	28.56(0.66)	22.99(0.04)	Positive	66.67%
		2	ND	ND	ND	28.61(0.81)	22.92(0.04)	Negative	
		3	34.1(NA)	31.41(NA)	30.14(1.09)	28.37(1.01)	22.97(0.1)	Positive	
	24	1	ND	31.2(0.75)	30.23(NA)	27.16(3.86)	22.95(0.12)	Positive	55.2%
		2	34.43(NA)	27.44(NA)	ND	28.95(0.73)	22.98(0.09)	Positive	
		3	34.66(NA)	31.67(NA)	29.8(NA)	28.67(1.02)	22.85(0.07)	Positive	
		4	33.76(NA)	28.58(4.84)	29.71(0.39)	27.7(1.42)	22.95(0.03)	Positive	
		5	ND	ND	ND	27.68(0.9)	22.83(0.1)	Negative	
		6	33.33(NA)	ND	30.21(NA)	28.19(0.49)	22.82(0.08)	Positive	
		7	33.5(NA)	30.48(NA)	ND	27.74(0.42)	22.62(0.21)	Positive	
		8	33.38(NA)	ND	ND	26.87(2.66)	22.92(0.02)	Inconclusive	
		9	32.37(NA)	ND	ND	27.85(0.38)	22.76(0.2)	Inconclusive	
		10	ND	ND	ND	27.49(0.44)	23.78(0.06)	Negative	
		11	ND	ND	ND	27.79(0.49)	23.86(0.13)	Negative	
		12	33(NA)	ND	33.03(NA)	26.81(0.38)	22.58(0.16)	Positive	
		13	ND	ND	32.58(NA)	26.75(0.33)	22.4(0.08)	Inconclusive	
		14	ND	ND	ND	27.9(0.38)	23.88(0.14)	Negative	
		15	ND	ND	ND	27.88(0.4)	23.9(0.13)	Negative	
		16	ND	ND	ND	26.93(0.4)	22.68(0.05)	Negative	
		17	34.22(NA)	ND	ND	26.9(0.15)	22.7(0.08)	Inconclusive	
		18	ND	ND	ND	27.06(0.42)	23.55(0.03)	Negative	
		19	ND	ND	ND	26.69(0.49)	23.35(0.01)	Negative	
		20	34.71(NA)	ND	ND	26.88(0.57)	23.16(0.19)	Inconclusive	
		21	ND	ND	ND	26.91(0.6)	23.05(0.13)	Negative	
		22	ND	ND	ND	27.1(0.47)	23.34(0.03)	Negative	
		23	ND	ND	ND	26.81(0.58)	23.01(0.25)	Negative	
		24	ND	ND	32.76(NA)	26.71(0.54)	23.09(0.13)	Inconclusive	
		25	ND	ND	ND	26.57(0.85)	22.96(0.17)	Negative	
		26	35.05(NA)	ND	32.5(0.22)	27.03(0.36)	23.63(0.16)	Positive	
		27	34.89(0.01)	ND	ND	27.37(0.26)	23.48(0.07)	Inconclusive	
		28	ND	ND	ND	27.41(0.42)	23.29(0.23)	Negative	
		29	ND	38.87(NA)	33.28(NA)	27.03(0.4)	23.19(0.09)	Inconclusive	
	36	1	ND	ND	ND	27.85(1.52)	22.93(0.09)	Negative	33.33%
		2	34.02(0.19)	31.54(1.1)	30.53(NA)	28.72(0.93)	22.78(0.03)	Positive	
		3	ND	ND	ND	28.2(1.13)	22.86(0.08)	Negative	
	48	1	ND	ND	ND	27.74(1.64)	22.84(0.02)	Negative	33.33%
		2	ND	ND	31.42(NA)	27(3.69)	22.68(0.15)	Inconclusive	
		3	ND	ND	ND	26.56(3.15)	22.75(0.06)	Negative	

313 GCE/ml (1.6 GCE/rxn)	1	1	31.71(1.12)	31.9(0.04)	28.74(0.41)	28.18(0.22)	22.88(0.12)	Positive	100%
		2	32.13(0.75)	30.57(0.77)	26.95(0.1)	28.36(0.2)	22.97(0.04)	Positive	
		3	32.39(1.83)	29.78(1.37)	26.52(2.99)	28.19(0.61)	23.31(0.11)	Positive	
	8	1	ND	30.62(2.75)	29.98(NA)	27.91(1.12)	23.16(0.02)	Positive	100%
		2	33.11(1.73)	31.26(0.01)	28.51(NA)	28.19(0.72)	23.23(0.14)	Positive	
		3	32.9(1.11)	29.8(2.68)	ND	27.92(1.37)	23.29(0.06)	Positive	
	16	1	ND	ND	31.21(NA)	28.7(0.64)	23.18(0.04)	Inconclusive	100%
		2	33.8(0.71)	ND	ND	27.33(1.92)	22.96(0.02)	Inconclusive	
		3	31.81(NA)	31.91(NA)	ND	28.09(1.35)	23.05(0.03)	Positive	
	24	1	ND	ND	ND	25.95(5.11)	22.88(0.08)	Negative	0%
		2	ND	ND	ND	26.7(3.7)	22.81(0.05)	Negative	
		3	ND	ND	ND	28.01(1.02)	22.81(0.11)	Negative	
	36	1	ND	ND	ND	25.96(5.06)	22.99(0.14)	Negative	0%
		2	ND	ND	ND	27.34(2.41)	22.66(0.09)	Negative	
		3	ND	ND	ND	26.42(3.76)	22.81(0.12)	Negative	
	48	1	33.73(NA)	ND	ND	28.17(0.44)	22.77(0.21)	Inconclusive	33.33%
		2	ND	ND	ND	27.34(0.89)	22.6(0.14)	Negative	
		3	ND	ND	ND	28.04(0.67)	22.77(0.07)	Negative	
78 GCE/ml (0.8 GCE/rxn)	1	1	33.46(1.1)	30.4(NA)	29.14(NA)	28.57(0.31)	23.53(0.06)	Positive	100%
		2	33.52(1.36)	29.61(0.98)	27.78(6.33)	28.05(0.58)	23.29(0.22)	Positive	
		3	31.4(2.4)	31.48(0.39)	29.58(2.25)	28.33(0.28)	23.41(0.07)	Positive	
	8	1	ND	32.76(NA)	ND	28.65(0.9)	23.66(0.06)	Inconclusive	100%
		2	33.34(1.08)	ND	ND	28.73(0.44)	23.57(0.1)	Inconclusive	
		3	34.02(NA)	ND	29.64(NA)	28.15(0.39)	23.36(0.17)	Positive	
	16	1	29.76(NA)	ND	ND	25.9(5.48)	23.17(0.1)	Inconclusive	66.67%
		2	32.4(1.9)	ND	ND	28.69(1.11)	23.44(0.07)	Inconclusive	
		3	ND	ND	ND	28.25(0.46)	23.35(0.04)	Negative	
	24	1	ND	ND	ND	28.37(1.62)	22.93(0.17)	Negative	0%
		2	ND	ND	ND	28.89(1.04)	23.15(0.16)	Negative	
		3	ND	ND	ND	28.4(0.43)	23.29(0.14)	Negative	
	36	1	ND	ND	ND	28.53(1.34)	23.11(0.05)	Negative	0%
		2	ND	ND	ND	28.88(0.44)	23(0.11)	Negative	
		3	ND	ND	ND	28.82(0.23)	23.31(0.1)	Negative	
	48	1	ND	ND	ND	28.62(0.72)	23.07(0.05)	Negative	0%
		2	ND	ND	ND	28.75(0.48)	23.29(0.08)	Negative	
		3	ND	ND	ND	28.1(0.28)	23.18(0.06)	Negative	

ND – not detected.
N – replicate number.
