# Supplementary material for: Implementation of a pooled surveillance testing program for asymptomatic SARS-CoV-2 infections in K-12 schools and universities
Source: eClinicalMedicine. 2021 Jul 17;38:101028. doi: 10.1016/j.eclinm.2021.101028 (PMC8286123; doi:10.1016/j.eclinm.2021.101028)
Supplement: Supplementary file 3 [file mmc3.pdf]

Supplementary Table 3. Representative comparison of Ct values of individual specimens and pools of 24. Pools of 24 samples were generated and tested for SARS-CoV-2 viral RNA. Pools with a single gene amplification were triggered for reflex to identify the individual positive samples(s) within the pool. Pools of 24 with a single positive sample identified were selected.

Pool Mean CT values (SD); N=3					Individual Mean CT values (SD); N=3				
**Pool NO.**	**MS2 Phage**	**N gene**	**Orf1ab gene**	**S gene**	**Individual NO.**	**MS2 Phage**	**N gene**	**Orf1ab gene**	**S gene**
02426781	26.83 (0.77)	29.6 1(2.08)	31.62 (0.65)	30.07 (1.40)	00209967	24.86 (0.12)	27.14 (0.20)	27.34 (0.24)	26.94 (0.38)
08146338	26.91 (0.99)	30.11 (0.79)	31.88 (1.26)	30.25 (2.39)	00056783	24.91 (0.72)	27.26 (0.30)	28.22 (0.11)	28.45 (0.52)
02343926	25.87 (0.41)	29.97(0.03)	29.26 (0.11)	29.90 (0.09)	02537656	28.72 (0.10)	27.58 (0.08)	28.17 (0.13)	27.43 (0.08)
02424449	27.38 (0.84)	30.24 (0.08)	30.57 (0.63)	30.43 (0.60)	00033197	25.04 (0.09)	27.61 (0.16)	26.99 (0.40)	26.58 (0.19)
02425412	26.45 (0.27)	30.87 (0.84)	33.60 (0.39)	32.31 (0.22)	00936516	24.72 (1.15)	28.58 (1.05)	27.08 (2.24)	21.71 (6.65)
02342504	25.48 (0.44)	31.59 (NA)	34.39 (0.92)	34.08 (NA)	02572290	24.34 (1.08)	28.58 (1.66)	28.82 (1.97)	25.94 (5.27)
08144478	29.55 (0.07)	32.42 (0.62)	33.15 (0.58)	32.49 (0.68)	00072563	25.44 (0.18)	28.87 (0.12)	29.26 (0.01)	29.80 (0.24)
02339381	27.11 (0.18)	30.42 (0.46)	31.39 (0.44)	31.00 (0.53)	02542540	27.10 (0.75)	28.94 (0.24)	28.25 (0.36)	27.96 (0.37)
02342514	25.88 (0.32)	30.61 (NA)	34.25 (0.30)	31.38 (0.22)	02570484	25.37 (0.66)	29.06 (0.32)	29.96 (0.35)	29.08 (0.44)
02343358	26.09 (0.53)	34.78 (NA)	33.43 (2.40)	38.20 (NA)	02601428	26.40 (0.26)	29.08 (0.61)	32.40 (0.41)	31.86 (0.50)
07211899	27.50 (1.88)	30.71 (0.13)	32.52 (0.16)	31.66 (1.62)	07211857	29.48 (0.85)	29.42 (0.37)	30.95 (0.33)	29.75 (0.50)
02423430	23.60 (0.64)	32.44 (1.18)	31.91 (0.94)	31.82 (0.82)	00314486	25.46 (0.22)	29.62 (0.03)	28.98 (0.18)	28.46 (0.20)
02339514	26.68 (1.24)	31.56 (NA)	36.01 (NA)	31.14 (NA)	02621847	26.19 (1.05)	29.79 (0.68)	30.57 (1.00)	28.09 (3.16)
02422747	31.42 (0.50)	34.08 (NA)	33.00 (0.35)	33.97 (1.18)	02571625	25.87 (0.36)	29.91 (0.05)	31.13 (0.08)	30.45 (0.06)
02382707	28.36 (0.52)	33.43 (0.04)	35.66 (0.92)	35.20 (NA)	00240737	24.50 (0.06)	29.93 (0.61)	30.58 (0.35)	29.73 (0.53)
02343089	30.79 (0.15)	33.01 (0.07)	33.42 (0.53)	34.65 (1.20)	00968143	27.38 (0.41)	30.22 (0.19)	29.69 (0.28)	30.56 (0.45)
02340249	28.81 (0.27)	32.09 (1.14)	34.96 (1.12)	34.78 (0.21)	00913706	25.84 (0.2)	31.01 (0.37)	30.97 (0.05)	31.23 (0.34)
02341926	27.67 (0.35)	34.25 (NA)	34.46 (NA)	34.88 (0.31)	00039323	24.84 (2.95)	31.09 (0.27)	30.19 (0.66)	28.43 (1.98)
02343186	28.65 (0.42)	33.06 (NA)	34.5 (NA)	35.59 (0.54)	02585218	25.84 (0.6)	31.16 (1.02)	31.97 (0.92)	32.26 (NA)
02340362	27.30 (1.12)	34.07 (0.20)	34.94 (NA)	34.04 (NA)	02583435	26.58 (0.21)	31.52 (0.34)	32.17 (0.25)	32.14 (1.09)
