# Supplementary material for: Implementation of a pooled surveillance testing program for asymptomatic SARS-CoV-2 infections in K-12 schools and universities
Source: eClinicalMedicine. 2021 Jul 17;38:101028. doi: 10.1016/j.eclinm.2021.101028 (PMC8286123; doi:10.1016/j.eclinm.2021.101028)
Supplement: Supplementary file 5 [file mmc5.pdf]

Supplementary Table 5. Outlier detection analysis table for October 31st (Halloween). The date with an outlier data is highlighted in green.

Date	Aggregate individual number	Aggregate positivity rate	Modified Z score	Modified Outlier?
10/1/2020	1558	6.42E-04	-0.30	No
10/2/2020	1614	0.00E+00	-0.67	No
10/3/2020	878	3.42E-03	1.33	No
10/6/2020	1541	6.49E-04	-0.29	No
10/8/2020	3634	8.26E-04	-0.19	No
10/9/2020	2551	0.00E+00	-0.67	No
10/13/2020	2119	0.00E+00	-0.67	No
10/15/2020	2280	3.07E-03	1.13	No
10/16/2020	4828	0.00E+00	-0.67	No
10/19/2020	2277	0.00E+00	-0.67	No
10/20/2020	3854	0.00E+00	-0.67	No
10/21/2020	1494	0.00E+00	-0.67	No
10/22/2020	3856	0.00E+00	-0.67	No
10/23/2020	4199	0.00E+00	-0.67	No
10/26/2020	532	0.00E+00	-0.67	No
10/27/2020	5158	0.00E+00	-0.67	No
10/28/2020	2867	0.00E+00	-0.67	No
10/29/2020	4939	4.05E-04	-0.44	No
10/30/2020	3388	2.66E-03	0.88	No
11/2/2020	2176	1.84E-03	0.40	No
11/3/2020	6131	9.79E-04	-0.10	No
11/4/2020	4992	2.80E-03	0.97	No
11/5/2020	8619	1.51E-03	0.21	No
11/6/2020	2403	4.16E-04	-0.43	No
11/9/2020	1205	4.98E-03	2.25	No
11/10/2020	7803	2.31E-03	0.68	No
11/11/2020	6731	4.75E-03	2.12	No
11/12/2020	5482	6.02E-03	2.86	No
11/13/2020	5181	9.65E-03	4.99	Yes
11/16/2020	1845	3.25E-03	1.23	No
11/17/2020	7291	1.92E-03	0.45	No
11/18/2020	7272	1.10E-03	-0.03	No
11/19/2020	4191	3.10E-03	1.15	No
11/20/2020	4840	2.69E-03	0.90	No
11/23/2020	870	1.15E-03	0.00	No

11/24/2020	2372	1.26E-03	0.07	No
11/25/2020	791	2.53E-03	0.81	No
11/30/2020	6283	2.23E-03	0.63	No
12/1/2020	5145	5.05E-03	2.29	No
