# Supplementary material for: Implementation of a pooled surveillance testing program for asymptomatic SARS-CoV-2 infections in K-12 schools and universities
Source: eClinicalMedicine. 2021 Jul 17;38:101028. doi: 10.1016/j.eclinm.2021.101028 (PMC8286123; doi:10.1016/j.eclinm.2021.101028)
Supplement: Supplementary file 6 [file mmc6.pdf]

Supplementary Table 6. Outlier detection analysis table for December 25th (Christmas) and December 31st (New Year). The dates with outlier data are highlighted in green.

Date	Aggregate individual number	Aggregate positivity rate	Modified Z score	Modified Outlier?
11/13/2020	5181	9.65E-03	1.83	No
11/16/2020	1845	3.25E-03	0.17	No
11/17/2020	7291	1.92E-03	-0.18	No
11/18/2020	7272	1.10E-03	-0.39	No
11/19/2020	4191	3.10E-03	0.13	No
11/20/2020	4840	2.69E-03	0.02	No
11/23/2020	870	1.15E-03	-0.38	No
11/24/2020	2372	1.26E-03	-0.35	No
11/25/2020	791	2.53E-03	-0.02	No
11/30/2020	6283	2.23E-03	-0.10	No
12/1/2020	5145	5.05E-03	0.64	No
12/2/2020	6490	9.24E-04	-0.43	No
12/3/2020	8937	2.46E-03	-0.04	No
12/4/2020	6235	1.60E-03	-0.26	No
12/7/2020	2040	1.96E-03	-0.17	No
12/8/2020	6808	1.62E-03	-0.26	No
12/9/2020	6610	1.97E-03	-0.16	No
12/10/2020	6841	2.05E-03	-0.14	No
12/11/2020	5170	4.06E-03	0.38	No
12/14/2020	1429	1.40E-03	-0.31	No
12/15/2020	7341	2.18E-03	-0.11	No
12/16/2020	4226	2.60E-03	0.00	No
12/18/2020	1224	1.47E-02	3.14	No
12/23/2020	563	7.10E-03	1.17	No
1/4/2021	2695	1.82E-02	4.04	Yes
1/5/2021	7570	7.66E-03	1.31	No
1/6/2021	3372	1.75E-02	3.86	Yes
1/7/2021	4791	2.40E-02	5.55	Yes
1/8/2021	4682	8.33E-03	1.48	No
1/12/2021	2976	6.05E-03	0.89	No
1/13/2021	516	1.55E-02	3.34	No
