# Supplementary material for: Implementation of a pooled surveillance testing program for asymptomatic SARS-CoV-2 infections in K-12 schools and universities
Source: eClinicalMedicine. 2021 Jul 17;38:101028. doi: 10.1016/j.eclinm.2021.101028 (PMC8286123; doi:10.1016/j.eclinm.2021.101028)
Supplement: Supplementary file 7 [file mmc7.pdf]

Supplementary Table 7. CT values of samples stored at room temperature or cycled through worst case shipping conditions using winter or summer excursion temperatures. A winter excursion consisted of cycling from -10°C for 8 hours, 18°C for 4 hours, -10°C for 2 hours, 10° C for 36 hours, and -10°C for 6 hours before testing. A summer excursion cycled from 40°C for 8 hours, 22°C for 4 hours, 40°C for 2 hours, 30°C for 36 hours, and 40°C for 6 hours before testing.

**Conditions**	**N**	**Mean CT values (SD; N=3)**			
		**MS2 Phage**	**N gene**	**ORF1ab**	**S gene**
Fresh	3	25.73 (0.44)	27.37 (0.24)	28.8 (0.23)	27.82 (0.3)
RT 5 days	10	26.47 (0.03)	29.03 (0.19)	28.27 (0.21)	30.02 (0.24)
RT 10 days	10	25.7 (0.14)	26.32 (0.15)	24.53 (0.52)	26.52 (0.10)
Winter	20	29.48 (1.79)	28.41 (0.46)	27.09 (1.03)	25.93 (0.94)
Summer	12	28.61 (1.07)	28.79 (0.57)	27.83 (0.85)	28.08 (1.23)
Fresh	3	25.62 (0.55)	26.87 (0.72)	27.2 (0.32)	27.58 (0.34)
RT 5 days	10	26.12 (0.14)	26.33 (0.07)	25.46 (0.01)	27.34 (0.08)
RT 10 days	10	25.14 (0.45)	28.30 (0.19)	27.04 (0.16)	28.76 (0.14)
Winter	15	28.25 (0.8)	28.52 (0.61)	27.57 (0.84)	28.01 (0.9)
Summer	8	28.02 (0.51)	28.76 (0.48)	27.66 (0.72)	26.01 (2.27)
