# Supplementary material for: Implementation of a pooled surveillance testing program for asymptomatic SARS-CoV-2 infections in K-12 schools and universities
Source: eClinicalMedicine. 2021 Jul 17;38:101028. doi: 10.1016/j.eclinm.2021.101028 (PMC8286123; doi:10.1016/j.eclinm.2021.101028)
Supplement: Supplementary file 8 [file mmc8.docx]

Supplementary Table 1. Summary of LOD preliminary range finding in individual specimen using known concentrations of ATCC VR-1986HK whole inactivated virus spiked into individual negative saliva. The stock concentration provided by ATCC was 4.2 x10^5^ GCE/µL. For each RNA extraction, 100µL of sample was used.

Supplementary Table 2. Summary of LOD preliminary range finding in pooled specimens using known concentrations of ATCC VR-1986HK whole inactivated virus spiked into individual negative saliva. The stock concentration provided by ATCC was 4.2x10^5^ GCE/µL. For each RNA extraction, 200µL of sample was used.

Supplementary Table 3. Representative comparison of Ct values of individual specimens and pools of 24. Pools of 24 samples were generated and tested for SARS-CoV-2 viral RNA. Pools with a single gene amplification were triggered for reflex to identify the individual positive samples(s) within the pool. Pools of 24 with a single positive sample identified were selected.

Supplementary Table 4. Surveillance results of all K-12 schools and universities tested over 12 weeks.

Supplementary Table 5. Outlier detection analysis table for October 31st (Halloween). The date with an outlier data is highlighted in green.

Supplementary Table 6. Outlier detection analysis table for December 25th (Christmas) and December 31st (New Year). The dates with outlier data are highlighted in green.

Supplementary Table 7. CT values of samples stored at room temperature or cycled through worst case shipping conditions using winter or summer excursion temperatures. A winter excursion consisted of cycling from -10°C for 8 hours, 18°C for 4 hours, -10°C for 2 hours, 10° C for 36 hours, and -10°C for 6 hours before testing. A summer excursion cycled from 40°C for 8 hours, 22°C for 4 hours, 40°C for 2 hours, 30°C for 36 hours, and 40°C for 6 hours before testing.
